# A Comparative Study of Endoscopic Ultrasound Fine-Needle Aspiration (EUS-FNA) and Endoscopic Retrograde Cholangiopancreatography (ERCP)-Based Brush Cytology for Tissue Diagnosis in Malignant Biliary Obstruction

**DOI:** 10.7759/cureus.30291

**Published:** 2022-10-14

**Authors:** Praveen Mathew, Prashant Kanni, Manoj Gowda, Chandrababu Devarapu, Jaseem Ansari, Achal Garg

**Affiliations:** 1 Gastroenterology and Hepatology, Vydehi Institute of Medical Sciences and Research Centre, Bangalore, IND; 2 Gastroenterology, Vydehi Institute of Medical Sciences and Research Centre, Bangalore, IND; 3 Gastroenterology, Deenanath Mangeshkar Hospital, Pune, IND

**Keywords:** fine needle aspiration, endoscopic ultrasound (eus), endoscopy ercp, malignant biliary obstruction, brush cytology

## Abstract

Background and objective

Patients with suspected malignant biliary strictures frequently undergo endoscopic retrograde cholangiopancreatography (ERCP)-based brush cytology and endoscopic ultrasound (EUS)-guided fine-needle aspiration (FNA) for establishing the diagnosis. The outcomes of these tests aid in the further management of the patient. A comparison of these two modalities in establishing the diagnosis is seldom reported. In light of this, we aimed to compare the diagnostic efficacy between ERCP-based brush cytology and EUS-FNA for tissue diagnosis in malignant biliary obstruction. Our study involved a retrospective audit of all patients admitted to the Vydehi Institute of Medical Sciences and Research Centre for EUS and ERCP from 2015 to 2019.

Methodology

A Comparative study was conducted in the Department of Medical Gastroenterology at the Vydehi Institute of Medical Sciences and Research Centre over a five-year period. A total of 77 subjects who presented during the study period with biliary obstruction based on clinical presentation with altered liver function test in an obstructive pattern and evidence of biliary obstruction in the form of stricture or pancreaticobiliary mass on cross-sectional imaging were included in the study. All the patients included in the study underwent EUS and ERCP.

Results

The majority of the patients in the study were in the fifth decade of life with a slight female predominance. The most common CT finding was a periampullary mass with common bile duct (CBD) stricture (59.7%). In the study, EUS-FNA was more sensitive than ERCP-based tissue sampling. The overall sensitivity was 90.63% for EUS-FNA and 65.63% for ERCP sampling. EUS-FNA was found to have diagnostic accuracy of 92.63% in comparison to 71.43% for brush cytology.

Conclusions

Based on our findings, EUS-FNA is superior to ERCP-based tissue sampling with excellent sensitivity and diagnostic accuracy. Performing EUS before ERCP in all patients with suspected malignant biliary obstruction would definitely improve diagnostic accuracy and thereby help in the management of such cases.

## Introduction

Benign and malignant lesions can lead to biliary tree obstructions. Differentiating malignant lesions from benign ones is an important step in the management of such cases [1]. Nearly 15-25% of the biliary strictures are benign, and distinguishing them from malignant strictures remains a challenge despite the availability of newer diagnostic imaging techniques, tissue acquisition, and cytological evaluation [2].

Endoscopic retrograde cholangiopancreatography (ERCP) is a widely used diagnostic and therapeutic intervention in the management of bile duct strictures. The two methods of obtaining tissue during ERCP include brushings of the biliary stricture for cytological evaluation and forceps for intraductal biopsies under fluoroscopic guidance. ERCP has a high specificity in diagnosing malignant strictures, but its sensitivity is low. Biliary brushing has a sensitivity of nearly 23-56% whereas biopsies have 35-65% sensitivity. A combination of both procedures has a sensitivity of 60-70% in the diagnosis of malignant strictures [3,4,5,6]. ERCP-based brush cytology is the most commonly used mode of tissue acquisition because of its feasibility and ease of access.

Endoscopic ultrasound-guided fine-needle aspiration (EUS-FNA) is a relatively newer modality of tissue acquisition. The fine-needle aspirate obtained by this procedure has been found to have a sensitivity in the range of 70-85% and specificity in the range of 90-97% in diagnosing malignancy [7,8,9]. The EUS-FNA is also favored for patients in whom biliary drainage/therapy is not warranted.

There are scant data comparing these methods in the literature, and hence the present study was conducted to compare both modalities of tissue acquisition in the diagnosis of malignant biliary obstruction.

This article was previously presented as a meeting abstract at the 61st Annual Conference of the Indian Society of Gastroenterology, Virtual Diamond Jubilee ISGCON 2020, December 19-20, 2020.

## Materials and methods

A comparative study was conducted in the Department of Medical Gastroenterology at the Vydehi Institute of Medical Sciences and Research Centre over a five-year period after obtaining ethical committee clearance from the Vydehi Institutional Ethics Committee - VIEC; EC REG NO: ECR/747/INST/KA/2015; approval number: VIEC/2018/app/208. The main aim of the study was to evaluate the efficacy of ERCP-based tissue sampling by brush cytology in comparison with EUS FNAC. The study was done to address the issue of underdiagnosing biliary malignancies and to improve diagnostic accuracy with EUS-guided tissue acquisition techniques. A total of 77 subjects who presented during the study period with biliary obstruction based on clinical presentation with altered liver function test in an obstructive pattern and evidence of biliary obstruction in the form of stricture or pancreaticobiliary mass on cross-sectional imaging were included in the study. Patients with features of cholangitis on evaluation and drainage of purulent material on ERCP were excluded from the study. All the patients included in the study underwent EUS and ERCP. Patients without suspicion of malignancy or those who underwent EUS but did not require ERCP were not included in the study.

Participants underwent EUS (Olympus Linear Echoendoscope GF UCT 180; Olympus Corporation, Tokyo, Japan)-guided FNA from focal bile duct mass or strictures or pancreatic mass or lymph nodes depending on the finding using a 22-G or 25-G needle. All FNAs underwent on-site cytological examination and the adequacy of the sample was confirmed by the cytopathologist. Additional FNA passes were also made depending on the adequacy. Tissue samples obtained during EUS-FNA were classified into one of the following categories: (1) malignant; (2) atypical, suspect malignant; (3) atypical, favor benign; and (4) benign. Samples that were labeled malignant and atypical and suspicious of malignancy were considered malignant.

ERCP (Olympus TJF 150)-based tissue sampling was done using the cytology brush (Boston Scientific RX Cytology Brush M00545000; Boston Scientific, Marlborough, MA). Cytology brushings were obtained using 10 to-and-fro brushings across the biliary obstruction. The brush was then smeared and adequacy was confirmed by the onsite cytopathologist. The final diagnosis for the study was based on the surgical or biopsy or EUS/ERCP findings.

## Results

In this study, the majority of subjects were in the age group of 51-60 years (40.3%), as seen in Table 1. The mean age of the subjects was 51.91 ± 10.3 years; 50.6% were females and 49.4% were males, as shown in Table 1.

**Table 1:** Age and gender distribution of subjects

		Count	%
Age	<30 years	4	5.2%
	31–40 years	3	3.9%
	41–50 years	28	36.4%
	51–60 years	31	40.3%
	61–70 years	7	9.1%
	>70 years	4	5.2%
Gender	Male	38	49.4%
	Female	39	50.6%

The most common location of stricture was the common bile duct (CBD), and the most frequent CT finding was periampullary mass (59.7%) as seen in Table 2.

**Table 2:** Location of stricture and CT findings CBD: common bile duct; CT: computed tomography

		Count	%
Location of stricture/CT finding	CBD stricture/chronic pancreatitis	7	9.10%
	CBD stricture/periampullary mass	46	59.70%
	CBD stricture/distal cholangiocarcinoma	9	11.70%
	Perihilar stricture/proximal cholangiocarcinoma	8	10.40%
	Perihilar stricture/Ca gallbladder	3	3.90%

As shown in Table 3, with ERCP brush cytology, 54.5% were malignant lesions, and 45.5% were benign lesions. On EUS-FNAC, 75.3% were malignant lesions, and 24.7% were benign lesions.

**Table 3:** Classification of lesions based on brush cytology and EUS-FNAC EUS-FNAC: ultrasound-guided fine-needle aspiration cytology

		Count	%
Brush cytology	Malignant	42	54.5%
	Benign	35	45.5%
EUS-FNAC	Malignant	58	75.3%
	Benign	19	24.7%
Final diagnosis	Malignant	64	83.1%
	Benign	13	16.9%

As shown in Table 4, among 64 subjects with malignancy, 65.6% were malignant in brush cytology and 34.4% were benign lesions (false negative). Among 13 subjects with benign lesions, 100% were benign in brush cytology. There was a significant association between brush cytology and final diagnosis. Brush cytology had a sensitivity of 65.63%, specificity of 100%, positive predictive value (PPV) of 100%, negative predictive value (NPV) of 37.14%, and diagnostic accuracy of 71.43%.

**Table 4:** Association between brush cytology and final diagnosis

		Final diagnosis			
		Malignant		Benign	
		Count	%	Count	%
Brush cytology	Malignant	42	65.6%	0	0.0%
	Benign	22	34.4%	13	100.0%
Parameter		Estimate		95% CI	
Sensitivity		65.63%		53.4, 76.08	
Specificity		100%		77.19, 100	
Positive predictive value		100%		91.62, 100	
Negative predictive value		37.14%		23.17, 53.66	
Diagnostic accuracy		71.43%		60.51, 80.31	

As shown in Table 5, among 64 subjects with malignancy, 90.6% were malignant in EUS-FNAC, and 9.4% were benign lesions (false negative). Among 13 subjects with benign lesions, 100% were benign in EUS-FNAC. There was a significant association between EUS-FNAC and final diagnosis. EUS-FNAC had a sensitivity of 90.63%, specificity of 100%, PPV of 100%, NPV of 68.42%, and diagnostic accuracy of 92.21%.

**Table 5:** Association between EUS-FNAC and final diagnosis EUS-FNAC: ultrasound-guided fine-needle aspiration cytology

		Final diagnosis			
		Malignant		Benign	
		Count	%	Count	%
EUS-FNAC	Malignant	58	90.6%	0	0.0%
	Benign	6	9.4%	13	100.0%
Parameter		Estimate		95% CI	
Sensitivity		90.63%		81.02, 95.63	
Specificity		100%		77.19, 100	
Positive predictive value		100%		93.79, 100	
Negative predictive value		68.42%		46.01, 84.64	
Diagnostic accuracy		92.21%		84.02, 96.38	

## Discussion

In the present study, we compared two methods used in the diagnosis of malignancy in biliary strictures. In the study by Oppong et al. [10], EUS-FNA had a higher sensitivity of 53% when compared to ERCP brush cytology, which yielded 29% sensitivity in diagnosing malignant tumors causing biliary obstruction. In a study by Weilert et al. [11], which involved diagnosing malignant biliary obstruction by the two diagnostic methods, it was observed that EUS-FNA had a sensitivity of 94% and diagnostic accuracy of 94% whereas ERCP brush cytology had a lower sensitivity of 50% and diagnostic accuracy of 53%. The sensitivity of EUS-FNA for pancreatic masses was 100% in comparison to ERCP-based tissue sampling, which was 38%.

In the study by DeWitt et al. [12], 24 patients with proximal biliary stricture with negative or inconclusive brush cytology underwent EUS-FNA. With EUS, a mass could be visualized including in the 13 cases where there was no lesion on cross-sectional imaging. EUS-FNA revealed malignancy in 17 out of 24 cases. The sensitivity, specificity, and PPV of EUS-FNA in the study were 77%, 100%, and 100% respectively.

In the study by Gress et al. [13], EUS-FNA was found to be better than ERCP. No false positive results were observed in the EUS-based technique and three patients who were diagnosed negative for malignancy in ERCP were further diagnosed as positive in EUS-FNA. The study by Rösch et al. [14] observed that the sensitivity and specificity of ERCP-based brush cytology were 46% and 100% while EUS-FNA had a sensitivity of 75% and specificity of 100%.

A study by Mohamadnejad et al. [15] found that tumor detection was superior with EUS in comparison with tri-phasic CT. The sensitivity of EUS-FNA for the diagnosis of cholangiocarcinoma was 73%. EUS also had 53% sensitivity and 97% specificity in the identification of unresectable tumors, making it an essential tool prior to major therapeutic decisions.

The findings of our study also revealed that ERCP brush cytology had a sensitivity of 65.3% while EUS-FNA had a sensitivity of 90.63%. Both diagnostic techniques had a very high specificity of 100%. The modest sensitivity and specificity of ERCP-based brush cytology could be attributed to the on-site analysis by the cytopathologist. The NPV of EUS-FNA was 68.42%, which was significantly more than that of the ERCP-based technique, which had an NPV of 37.14%. The findings of our study correlated with the majority of other studies in the literature and indicated that the EUS-FNA technique is definitely a better modality in diagnosing malignant biliary obstructions. In our study, both malignant and atypical findings suggestive of malignancy were considered as definite malignancy, which could have been one of the reasons for the high sensitivity and positive predictive value.

Our study has a few limitations. The study comprised a mixed cohort of patients with proximal and distal biliary structures resulting from multiple etiologies. Newer diagnostic modalities like cholangioscopy-assisted tissue acquisition, intraductal ultrasound-tissue acquisition, and probe-based confocal laser endomicroscopy (pCLE) were not used. Also, newer tissue diagnostic modalities like fluorescence in situ hybridization (FISH) and molecular analysis of cell-free cytology brush were not used in our study, which would have improved the diagnostic accuracy of ERCP brush cytology. The gold standard method for diagnosis of malignant pathology is the histological evaluation of surgical specimens, which could not be done in our study, as most of the patients had advanced disease and underwent palliative procedures; only a few patients underwent definitive surgery.

## Conclusions

Among patients with biliary obstruction, ERCP-based brush sampling is a good technique for tissue acquisition with moderate sensitivity and high specificity. However, EUS-FNA is found to be superior to ERCP-based tissue sampling with excellent sensitivity and diagnostic accuracy. Performing EUS before therapeutic ERCP in all patients with suspected malignant biliary obstruction would definitely improve diagnostic accuracy and thereby help in the management of such cases.
